# Computed tomography-guided paravertebral doxorubicin injection for refractory pain in patients with spinal metastases: Two case reports

**DOI:** 10.1097/MD.0000000000018939

**Published:** 2020-01-31

**Authors:** Fan Lu, Qing Zhong, Jie Tian, Kexian Zhang

**Affiliations:** a Department of Anesthesiology, Sichuan Cancer Hospital & Institute, Sichuan Cancer Center, School of Medicine, University of Electronic Science and Technology of China, Chengdu; b Department of Anesthesiology and Pain Medicine, Jianyang People's Hospital, Jianyang, Sichuan, China.

**Keywords:** computed tomography guidance, four-step analgesia, interventional therapy, refractory cancer pain

## Abstract

**Rationale:**

Diagnosing and treating refractory cancer pain have become standardized and effective procedures with guidance from the Expert Consensus on Refractory Cancer Pain released in 2017 by the Committee of Rehabilitation and Palliative Care of China. Doxorubicin has been used for perineural injection in the treatment of chronic non-cancer pain owing to its retrograde sensory ganglion resection effect. Our study reports a new fourth-ladder treatment for cancer pain: CT-guided paravertebral doxorubicin injection for patients with refractory cancer pain caused by paraspinal metastasis.

**Patient concerns:**

A 48-year-old female and a 47-year-old male patients suffered from refractory cancer pain over the past months. They had both undergone surgical tumor resection, chemotherapy, and precision radiotherapy but result in limited analgesic effect. The daily oral morphine dosage was around 60 to 100 mg and rescue analgesic methods had been used at the time.

**Diagnoses:**

Refractory cancer pain in 2 patients with renal cancer and hepatobiliary adenocarcinoma.

**Interventions:**

The patients both received computed tomography (CT)-guided 1 mL of 0.5% doxorubicin paravertebral injection at each affected nerve root segments.

**Outcomes:**

The Visual Analog Scale and Douleur Neuropathique four Questions were used for 6-month follow-up, and the analgesic requirement was also recorded. The patients enjoyed satisfactory analgesia for up to 6 months without adverse reaction. In addition, the oral opioid analgesic doses were significantly reduced after the neurolytic block.

**Lessons:**

The CT-guided paravertebral doxorubicin injection was an effective fourth-step analgesic interventional technology that allowed our 2 patients with refractory cancer pain to maintain satisfactory analgesia. This analgesia method taken at an appropriate stage, according to the latest analgesic concept, results in good analgesia and opioid use reduction. Also, with the imaging guidance, only a small amount of neurolytic agent is needed to achieve analgesia in a precise and safe way.

## 1 Introduction

Refractory cancer pain presents diagnostic, management, and social challenges. In 2017, the Committee of Rehabilitation and Palliative Care of China published the first Expert Consensus for Refractory Cancer Pain. In this consensus, refractory cancer pain is defined as “medium or severe pain caused by cancer or other related factors during anti-cancer treatment that cannot be effectively relieved after 1 to 2 weeks of standardized medication, and/or whose treatment causes intolerable side effects”.^[1]^ In addition, this consensus also includes diagnostic criteria and treatment guidelines. The fourth-step analgesia techniques include neurolytic block, patient-controlled analgesia, and implantable drug delivery systems, listed as the main methods for refractory cancer pain management. The four-step analgesic concept allows pain practitioners to skip the steps in the treatment of cancer pain, or to choose a 2-way path starting from a high step and returning back to a low step according to the pain condition.^[2]^

Doxorubicin has been used as a neurolytic agent for para neural injection based on its retrograde sensory ganglion resection effects.^[3,4]^ In clinical practice, doxorubicin injections are mainly used for neuropathic pain. Reports indicated that para neural injection of doxorubicin could effectively relieve pain in patients with trigeminal, intercostal, or post-herpetic neuralgias (PHN).^[5–7]^ However, few reports have used doxorubicin injection as a the fourth-step analgesia method to control refractory cancer pain. In another study, we investigated 1050 patients with cancer and found that the incidence of neuropathic pain was about 36.2% among them, and it increased to 73.3% among those with pain.^[8]^ Postoperative nerve injury, rib or paravertebral metastases, and tumor invasion are common causes of somatic neuropathic pain in cancer patients. Therefore, we hypothesized that target doxorubicin injection can also relieve somatic neuropathic pain in those patients. In our report, 2 patients with paravertebral metastases underwent computed tomography (CT)-guided paravertebral doxorubicin injection according to the four-step analgesia model; beneficial therapeutic effects were obtained in both patients and the amount of opioid analgesics used was reduced.

## 2 Case report

### 2.1 Case one

**Figure 1:**
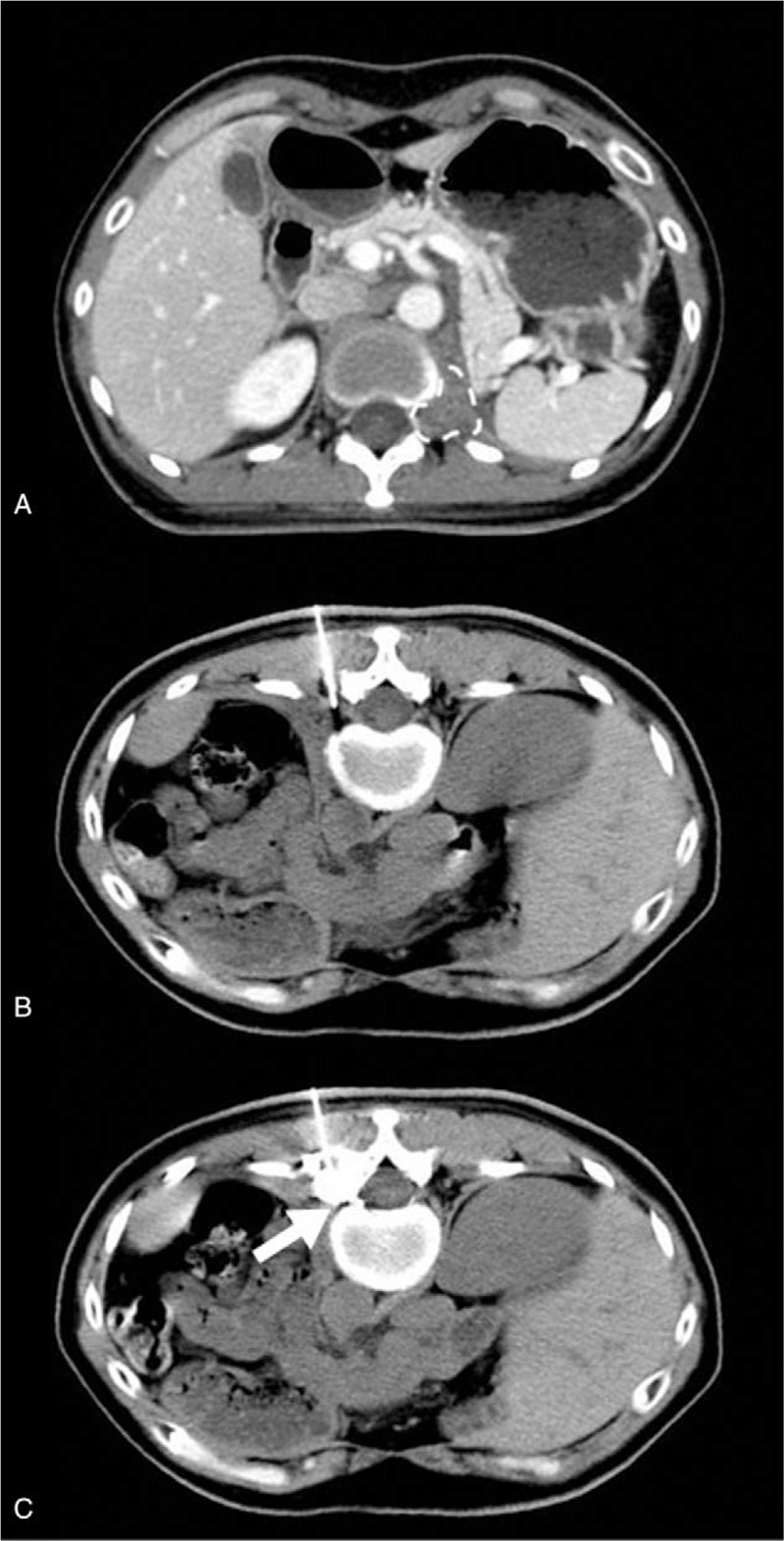
Computed tomography (CT) guided paravertebral doxorubicin injection: (A) CT image revealing the metastasis in the vertebral and intervertebral T12 foramina (dotted line area); (B) the inserting target was located near the left intervertebral foramen of T12-L1 level; (C) CT image confirming the contrast agent diffused through the intervertebral foramen into the dorsal epidural space.

A 48-year-old woman was admitted to our hospital for refractory cancer pain at the left side of the lower back caused by renal cancer. She was initially diagnosed as having “invasive urothelial carcinoma of the kidney” and had undergone left nephroureterectomy 1 year before. However, 6 months after the operation, she felt pain in the left lower back and a CT examination revealed a metastasis in the ipsilateral paravertebral space at T12 (Fig. 1A). During the next 5 months, she received 2 cycles of chemotherapy with gemcitabine (1400 mg/day on days 1 and 5) and oxaliplatin (200 mg/day on day 2) every 21 days, and 55-Gy external beam radiation therapy. However, the treatment did not completely eliminate the metastasis in the vertebral and intervertebral T12 foramina. The patient complained of persistent, tingling and burning pain on the lower left back and abdomen, and the average visual analog scale (VAS) score was 6 points. In addition, the Douleur Neuropathique four Questions (DN4) questionnaire was used to evaluate neuropathic pain, and her initial score was 5 points. The patient had received long-term analgesic medication with pregabalin (300 mg/day), oxycodone (escalated from 20 to 100 mg/day), and intramuscular dezocine (5 mg) for breakthrough pain, but was not obtaining sufficient analgesic effects. On the contrary, the patient gradually developed dizziness, nausea, anorexia, and constipation. We addressed the next pain control strategy in a multidisciplinary consultation with pain specialists, oncologists, and radiation therapists. Chemoradiotherapy and surgical treatment were considered inapplicable for the patient at this stage. Thus, based on the principle of the four-step analgesia ladder, we offered 2 minimally invasive intervention options for the patient as follows: intrathecal morphine pump placement and neuraxial chemical neurolysis. The patient chose the second option due to the high cost of the intrathecal pump surgery. Before the invasive procedure, we performed a paravertebral block at T12 under ultrasound guidance with 5 ml of 1% lidocaine, and the patient reported immediate pain relief after the block that lasted for 6 hours. Therefore, we administered a left paravertebral doxorubicin injection at T12 under CT guidance.

The Ethics Committee approved the treatment protocol. We informed the patient that the procedure was being applied for the management of the refractory cancer pain and she freely signed an informed consent form.

The patient was placed in a prone position with a thin pillow under the abdomen to keep the back parallel to the CT gantry, and was monitored by pulse oximetry and noninvasive blood pressure. An intravenous line was set up with IV balanced fluids. We obtained the puncture target image (left intervertebral foramen between T12 and L1) by CT scanning, and marked the puncture site under infrared positioning. We advanced a 22-G needle toward the target via CT guidance, and injected 1 mL of iohexol (Omnipaque) followed by negative aspiration. After confirming the contrast spread into the target intervertebral foramen, we injected 1% lidocaine in 3 mL to test the effect. The patient reported pain relief without any discomfort during the 10-minute observation period; thus, we injected 1 mL of 0.5% doxorubicin into the intervertebral foramen. We withdrew the needle while injecting a small amount of normal saline (Fig. 1).

The patient reported significant pain relief in the lower left back around the T12 dermatome, and we gradually decreased the analgesics to oxycodone 20 mg/day and pregabalin 150 mg/day during the following days. She achieved complete pain relief 2 weeks after the injection and stopped using any analgesics. At the first month follow-up, the patient reported slight numbness on the skin of the T12 nerve innervation area and she scored 2 points on the DN4 scale. During the following 5 months, the patient underwent a course of radiotherapy for the residual metastasis and no pain was reported throughout the anti-cancer treatment.

### 2.2 Case two

**Figure 2:**
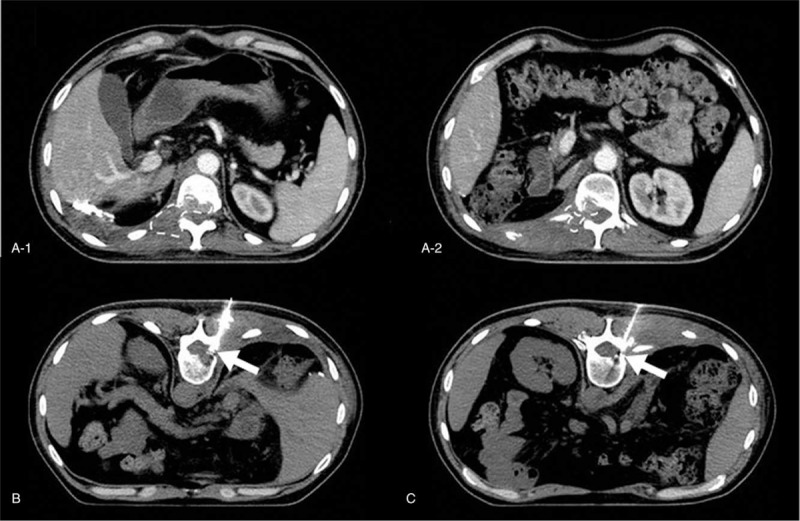
Computed tomography (CT) guided paravertebral doxorubicin injection: (A) CT image revealing the metastasis in the T11–12 paraspinal and thecal sac area (dotted line area) (A-1: T11, a-2: T12); (B, C) CT image confirming the correct inserting targets at the right intervertebral foramen of T11–12 and T12-L1 level respectively, the arrow points to the contrast agent.

A 47-year-old male patient was admitted to the pain department due to cancer pain in the right lower abdomen and inguinal area. He was diagnosed as having hepatobiliary adenocarcinoma with retroperitoneal metastasis. He had received multiple anti-tumor treatments during the past 3 years, and had had a pain in the right side of the abdomen for 8 months. Imaging revealed enhanced soft tissue thickening around the right paraspinal site at T11–12 (Fig. 2A). Thus, the patient underwent paravalvular palliative radiotherapy at a dose of 25 Gy. However, the analgesic effect of radiotherapy was negative. He complained about a persistent dull, burning pain and soreness accompanied by paresthesia in the T11–12 dermatome, with an average VAS score of 6 points (8 points at its worst, 3 points at its least), and an average DN4 of 6 points. The patient received oxycodone (120 mg/day) and pregabalin (300 mg/day), but his pain scores were not reduced as a result of the treatment. In addition, his sleep quality was severely affected and he could not even lie on his right side. According to the diagnostic criteria of the expert consensus, we diagnosed the patient as having refractory cancer pain. Then, this case was introduced through our multidisciplinary channel, and further analgesic strategy for this patient was developed by multidisciplinary decision making and a comprehensive informed consent. We decided that the neuraxial neurolysis could be one of the best next analgesic approaches for him because the metastasis was indenting the thecal sac at the T12 level, which is a potential contraindication for intrathecal pumps. In addition, further palliative radio- and chemotherapy or surgery were infeasible according to the experts’ decisions. However, we are still concerned about the outcome of the neurolytic blocks because the tumor invades around the block target. Therefore, an ultrasound-guided paravertebral block with 1% lidocaine in 5 mL was first performed. Fortunately, the patient responded well to the test block; hence, he was further administered a CT-guided paravertebral doxorubicin injection.

Therefore, we obtained the approval from the ethics committee and the patient's informed consent, and performed a minimally invasive operation on the T11 and T12 nerve root segments. We determined the operation target based on CT scan images. We advanced 2 needles toward the T11 and T12 intervertebral foramina and tested a 3-mL analgesic dose (1% lidocaine). After 10 minutes, the patient reported pain relief and we proceeded to slowly inject 1 mL of 0.5% doxorubicin into the 2 sites (Fig. 1).

On the first day after the operation, the patient reported significant relief from the right lower back pain and approximately 50% pain relief in the lower abdomen and groin. In addition, he could now sleep on his right side, and turning over was not aggravating the pain. One month later, the pain was relieved completely, and we detected hypoesthesia at the T11 and T12 dermatomes (DN4, 2 points). The pregabalin was reduced to 150 mg/day, and the use of oxycodone was stopped. In the second month after the operation, the patient received a course of chemotherapy for the cervical lymph node metastasis. The patient felt pain again (VAS, 3 points; DN4, 4 points) in the right lateral position at the 4th month, and we increased the pregabalin to 300 mg/day, which effectively controlled the pain.

## 3 Discussion

Paravertebral doxorubicin injection for analgesia is not an experimental procedure that is commonly used in clinical practice; this technique is mainly used for patients with neuropathic pain in several pain management centers. Kato et al first reported the clinical application of doxorubicin for the treatment of neuropathic pain in the early 1990s,^[9]^ and the agent can now be used as a neurolytic agent for neurotomy. In that report, the authors described the clinical use of 1% to 2% doxorubicin to treat patients with trigeminal and PHN, and the patients achieved significantly sustained pain relief and were able to go back to work. Based on the initial pain treatment experience with doxorubicin, the agent has been applied to treat conditions such as trigeminal and PHN and other chronic pains. In the early PHN treatment reports, the effective rate for the doxorubicin injection was about 67.7%,^[10]^ and this number has improved with the application of visualization technologies. Studies have reported significant reductions in postoperative VAS scores and increased patient-related quality of life and sleep time with paravertebral doxorubicin injections under the guidance of C-arm or CT.^[7,11,12]^ For cancer pain treatment, one report showed a 0.5-mg doxorubicin injection for the brachial plexus under CT guidance resulted in pain improvement without damage to the motor function of the upper limb and neck.^[13]^ In our cases, we used 0.5% doxorubicin for intervertebral foramen injections in 2 patients with refractory cancer pain. Both of them reported rapid pain reduction in the target areas, and achieved the best pain belief during 2 weeks and 1 month after injection. The analgesic effects lasted up to 4 to 5 months with a decrease in analgesics usage and daily life improvement.

Ethanol and phenol are the 2 most common neurolytic agents presently used as neurolytic blocks, and both demonstrate a non-selectively destructive effect in Wallerian degeneration.^[14,15]^ Unlike ethanol and phenol, doxorubicin can selectively conduct neuronal degeneration through retrograde axoplasmic transport to the dorsal root ganglia after para neural injection, while the motor roots might be well preserved.^[16]^ After nerve injection, doxorubicin accumulates in the ganglion and preferentially localizes in the cells nuclei and nucleoli, causing neuronal destruction by inducing chromatin condensation, nuclear eccentricity, and nuclear membrane disappearance.^[17,18]^ In addition, doxorubicin can also cause structural changes on the nuclear pore complex, leading to protein synthesis disorders and ultimately destroying the sensory neurons.^[19]^ We noted that these findings are mainly based on the in vivo results obtained from animal models; thus, we are always cautious in the application of doxorubicin injection to our patients. To avoid any possible motor impairment, doxorubicin should not be injected into motor nerves such as brachial plexus and sciatic nerve. In addition, both the thoracic and abdominal musculatures take part in respiratory movements. It is necessary to minimize the number of target segments when administering paravertebral injection. In our patients, the pain intensity and its characteristics improved significantly after paravertebral 0.5% doxorubicin injections according to VAS and DN4 evaluations, and we did not observe any motor dysfunction or other adverse reactions in our patients.

The amount of opioid used daily by a patient should not be used as a measure to decide whether the patient should receive minimally invasive analgesia; instead, the overall evaluation should confirm the presence of refractory cancer pain. According to the latest expert consensus, the diagnosis can be made when the following criteria are met:

1) pain scores remain ≥ 4 points and/or breakthrough pain occurs ≥ 3 times/day;
2) the pain cannot be relieved after 1 to 2 weeks of guideline recommend opioids and/or adjuvant analgesic treatment, and/or the treatments are causing intolerable side effects.^[1]^

We diagnosed the 2 patients in our report as presenting refractory cancer pain before simply increasing the opioids doses. Following these recommendations, the refractory cancer pain can be detected early, and minimally invasive analgesia should be introduced, especially when conventional analgesia strategies fail to reach satisfactory outcomes. In our report, the prognosis was favorable and the pain in patients was alleviated after they were administered paravertebral injection. Thus, they no longer needed any opioids during the following months.

Interventional pain management approaches have been important components of multimodal analgesia, and the timing of interventional treatment is a key factor for prognosis. Approximately 20% of patients with cancer pain fail to achieve significant relief from the three-step analgesic ladder.^[20]^ In addition, some patients are unable to tolerate opioid-related adverse effects (nausea, vomiting, constipation, or sedation) and are forced to suspend the medication, resulting in inadequate analgesia.^[21]^ These situations prompted the advanced fourth-step on the World Health Organization analgesic ladder, which includes mainly interventional therapies.^[22]^ In patients with refractory cancer pain, guidelines recommend the application of fourth-step intervention approaches, including epidural infusion, nerve block, neurolytic blockade, radiotherapy, and surgery.^[23,24]^ However, the indication for intervention approaches is unclear and varies in different clinical practices due to lacking definitions and diagnostic criteria for refractory cancer pain. Therefore, some patients are not optimally treated. The Expert Consensus on Refractory Cancer Pain, which regulates the diagnosis and treatment of refractory cancer pain, has reduced the magnitude of this problem. In our patients, we detected the refractory cancer pain after 1 to 2 weeks of failed attempted opioid rotation by guideline criteria in both cases, so we applied the neurolytic techniques early, which resulted in favorable analgesic effects. We did not conduct a controlled study for the paravertebral cancer pain because of less number of cases and ethical considerations, which are inevitably limitations of this report. In addition, the anti-tumor therapy and tumor progression during the perioperative period may also affect the evaluation of the analgesic effect of doxorubicin injection, which may be worthy of further study in refractory cancer pain management.

## Author contributions

**Conceptualization:** Jie Tian, Kexian Zhang.

**Resources:** Qing Zhong.

**Writing – original draft:** Fan Lu.

**Writing – review & editing:** Jie Tian, Kexian Zhang.
